# Design and syntheses of 4-aminoquinoline-thiazolidinone hybrids as anti-protozoal agents

**DOI:** 10.3389/fchem.2026.1892807

**Published:** 2026-09-07

**Authors:** Gul Naz Fatima, Sarvesh K. Paliwal, Shailendra K. Saraf

**Affiliations:** 1 Division of Pharmaceutical Chemistry, Faculty of Pharmacy, Babu Banarasi Das Northern India Institute of Technology, Lucknow, Uttar Pradesh, India; 2 Department of Pharmacy, Banasthali Vidyapith, Tonk, Rajasthan, India

**Keywords:** 4-aminoquinoline, 4-thiazolidinone, *in-vitro*, *in-vivo*, molecular docking, molecular hybridization

## Abstract

**Introduction:**

Molecular hybridization is an effective approach employed to synthesize molecules that possess the ability to function as dual inhibitors, thereby addressing the issue of growing resistance. Resistance by the malaria parasite is one such example. Therefore, the study aims to develop hybrid compounds consisting of 4-aminoquinoline and 4-thiazolidinone, utilizing molecular docking for sorting, followed by synthesis and evaluation of their anti-malarial potential.

**Methods:**

The molecules were selected on the basis of affinity from the computational study. To strengthen the *in silico* docking, a 200ns molecular dynamics simulation study was also performed. The title compounds, 7-18, were synthesized in a three-step reaction, involving non-polar solvent addition reaction resulting in heterocyclization. The synthesis was followed by characterization of all the synthesized compounds, and they were further subjected to biological screening through *in-vitro*, and the selected actives to *in-vivo* evaluation. An acute toxicity study was also conducted.

**Results:**

The compounds exhibited promising action, with five compounds, 8, 9, 14, 15, and 18, demonstrating efficacy comparable to the reference drug in the *Pf*-DHFR enzyme inhibition assay. Four of the active compounds selected for the *Plasmodium berghei* murine model displayed excellent activity, with percentage parasitaemia inhibition to the extent of 84%, and mean survival time up to 27 days. The MD simulation study showed the stability of the ligand-protein complex up to 200 ns. The *in silico* ADMET prediction, along with acute toxicity testing of the most active compound, showed no major toxicity.

**Discussion:**

The study concludes the hybrid compounds to be promising antimalarial agents, and the scaffold may be a good lead for the development of next-generation antimalarials.

## 1 Introduction

The life cycle of the parasite in the human host begins when the sporozoites are introduced into the bloodstream during a blood meal by infected female Anopheles mosquitoes (Bousema et al., 2014; Rosado-Quiñones et al., 2024). These are subsequently transported to the liver, reach the pre-erythrocytic schizont phase, mature to disseminate merozoites, followed by invasion and proliferation (Farrow et al., 2011). However, due to restricted capacity for *de novo* amino acid synthesis, the plasmodium depends on the hemoglobin of the host’s erythrocytes (Renar et al., 2016). In this process, the parasite breaks down significant quantities of hemoglobin into free heme and globin peptides through the action of plasmodium peptidases, and subsequently hydrolyzes the peptides into amino acids for use. The free heme is harmful as it produces reactive oxygen species that induce oxidative stress, resulting in DNA and membrane damage to the parasite, along with blockade of the peptidases, which further limits the breakdown of hemoglobin and the availability of amino acids. This explains why the parasite breaks down the heme into hemozoin, a harmless substance (Jones et al., 2015). Hemozoin is released into the host’s bloodstream, functioning as a plasmodium pathogen-associated molecular pattern (PAMP) that is recognized by receptors on immune cells (Matsuda et al., 2023). It also binds to toll-like receptor 9 (TLR9), thereby augmenting the host’s innate immunological response to malarial DNA. Hemozoin stimulates the NLRP3 inflammasome complex (Wang et al., 2022), either through Syk and Lyn tyrosine kinases or directly via DNA-complexed hemozoin released from the phagosome, leading to increased production of interleukin 1*β* (Gowda, 2007). Consequently, hemozoin induces an immuno-stimulatory response.

The established drugs for treatment of malaria are of the quinoline (Kapishnikov et al., 2021), and artemisinin (Hanboonkunupakarn et al., 2022; Lawrence et al., 2024) classes. The quinolines, which are further divided into aminoquinolines and aminoalcohols, act particularly by obstructing the process of heme detoxification. These drugs target the erythrocytic stage of parasite life cycles (Henry et al., 2006). They form a drug-hematin complex, which impairs hematin sequestration and destruction, causing hematin buildup and toxic effects on the parasite (Hisamatsu et al., 2025; Stocks et al., 2002). The basic amino group connected as a side chain to the 4-AQ moiety traps the drug into the parasite’s acidic digestive vacuole (DV), sustaining elevated concentrations of the drug (Yearick et al., 2008). Also, studies report the significance of AQ in targeting the enzyme dihydrofolate reductase (DHFR), establishing them as novel *Pf*-DHFR inhibitors (Sahu et al., 2016).

**FIGURE 1:**
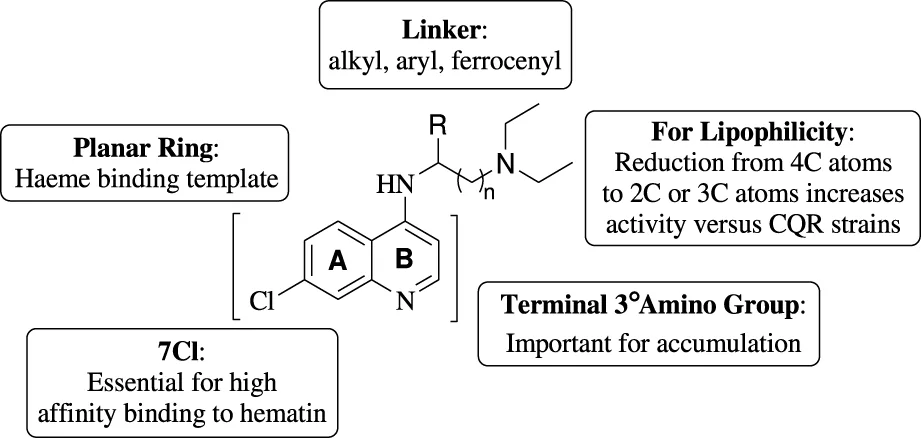
Structure-activity relationship of 4-aminoquinoline.

However, in recent decades, drug-resistant parasite strains have limited the choice of drugs utilized for treatment of the disease (Ejigu and Endalifer, 2023; Ezugwu et al., 2020; Fathoni et al., 2024). Studies suggest various modifications to the AQ scaffold (Shreekant and Bhimanna, 2016) that may help overcome resistance. These comprise modifications on the N-alkylamino side chain (Kondaparla et al., 2017), modifications on the third position of the ring B (Mushtaque, 2015), or modifications on the 4-AQ nucleus (Foley and Tilley, 1998; Figure 1). Further, the resistance may be due to reduced uptake, enhanced efflux of the drug, or a combination of both (Vandekerckhove and D’hooghe, 2015; Wicht et al., 2020), or mutations in the genes that encode the transport proteins located in the membrane of the DV of the parasite. As there are several reasons involved in resistance development (Kondaparla et al., 2017), a promising strategy could be a multi-targeted therapy (Bamigboye et al., 2026; Klein, 2013), in conjunction with some chemo-sensitizing agent (CSA). The CSAs exhibit sufficient properties to be considered as reversal agents in antimalarial treatment. They inhibit the enzymatic dealkylation of chloroquine while preserving its lipophilicity, hence enhancing its antimalarial efficacy (Rojas and Kouznetsov, 2011). The thiazolidin-4-ones (4-THZ) (Tripathi et al., 2014), along with other nitrogen-containing heterocyclic scaffolds, have been well-established for their role as CSA (Andrews et al., 2010; Ruiz et al., 2011). The hybrids thus obtained offer some advantage in overcoming resistance, along with an improvement in the biological potential (Pingaew et al., 2014).

In continuation of our work, the study reports the design of molecules and their synthesis involving a molecular hybridization strategy with the use of CSA. The synthesized compounds were characterized and tested for their anti-malarial efficacy, both *in-vitro* and *in-vivo*.

## 2 Materials and methods

### 2.1 Computational study: molecular docking

The malaria target protein crystal structures with their co-crystal ligand, having crystal resolution and R values below 3 Å and 0.25, respectively, were retrieved from the RCSB Protein Data Bank (PDB).

The Protein Preparation window of the Maestro4 graphical user interface of the Schrodinger application (Madhavi Sastry et al., 2013) was used to prepare the protein structures. Hydrogens were added to all atoms in the system, along with formal charges and bond orders for hetero-groups. The structures lacking side-chain atoms were predicted using the Prime refinement module. To minimize steric incompatibilities that might have existed in the original PDB structures, a quick relaxation was achieved using the Impact Refinement module employing the OPLS-2005 force field. When the energy converged, or the RMSD hit a maximum cutoff of 0.30 Å, the minimization was complete. The ligands were constructed using ChemDraw Ultra 7.0 and LigPrep 3.3 (v25111), and the Epik module (Shelley et al., 2007). All the molecules were subjected to conformational sampling using the ConfGen search method, and conformers were generated using ConfGen in conjunction with the OPLS 2005 force field. Duplicate poses were removed if the RMSD was less than 1.0 Å. A distance-dependent dielectric constant of four and a maximum relative energy differential of 10 kcal/mol were used as default settings during the preparation.

The re-docking trials confirmed the validity of the docking procedure. Glide docking (Friesner et al., 2004), Induced Fit Docking (IFD) (Sherman et al., 2006), and Quantum Polarized Ligand Docking (QPLD) (Cho et al., 2005) were employed in both SP and XP modes. The study assessed the energy parameters in terms of hydrogen bonding, hydrophobic interactions, *pi–pi* stacking, and electrostatic interactions, and represented them as glide scores. The protein and the docking method were selected based on the RMSD of the protein-ligand complex and the glide scores. Accordingly, four target proteins, 3LT0, 3BWK, 1LF2, and 3UM5, were selected. The Discovery Studio Client 2021 was used to visualize the ligand’s two-dimensional and three-dimensional docked conformations within the protein target.

### 2.2 Computational study: molecular dynamics

The molecular dynamics (MD) study of the highest binding ligand (8) was performed in order to determine the stability of the protein-ligand complex. The MD simulation study of the ligand 8-receptor complex was performed using the GROMACS 2020 package and the CHARMM-36M force field. The study increases the reliability of in *in silico* studies as this explains the actual conditions of the ligand and protein in physiologically simulated conditions.

### 2.3 ADMET prediction

Poor ADME-absorption, distribution, metabolism, and excretion profiles cause most drug candidates to fail clinical trials, thereby increasing the drug development costs. Early pharmacokinetic profiling of drug candidates saves time and resources, accelerating the development process. StarDrop software (Attwa et al., 2024), OSIRIS Property Explorer (Dong et al., 2018; Klebe, 2000), and the LAZAR (Cheng et al., 2012; Yang, 2010) programs were used for the prediction.

### 2.4 Chemistry

The chemicals and reagents were procured from Merck (Sigma-Aldrich), Germany, and T.C.I., Japan, and S.D. Fine Chemicals, India, and pre-coated TLC sheets were purchased from Merck, Germany. Solvents were of reagent grade and were purified and dried using standard procedures. The melting points were determined by the open capillary method and are uncorrected. The IR spectra were recorded on a Bruker FTIR, ALPHA-II (Eco-ATR) spectrophotometer (Bruker, Germany), and values are expressed in cm^-1^. ^1^H-NMR and ^13^C-NMR spectra were recorded on Bruker Avance-400, FT-NMR spectrometer (Bruker, Germany) at 400 MHz, and the chemical shifts are reported in parts per million (δ value), taking TMS (δ 0 ppm of ^1^H NMR) as the internal standard. The mass spectra were recorded on Waters UPLC-TQD Mass Spectrometer instrument (Waters Corporation, United States) using LC-ESI or APCI-MS Technique. Elemental analysis was performed on Perkin Elmer- 2400, Series-II Analyzer (Waltham, Massachusetts, United States).

**SCHEME 1:**
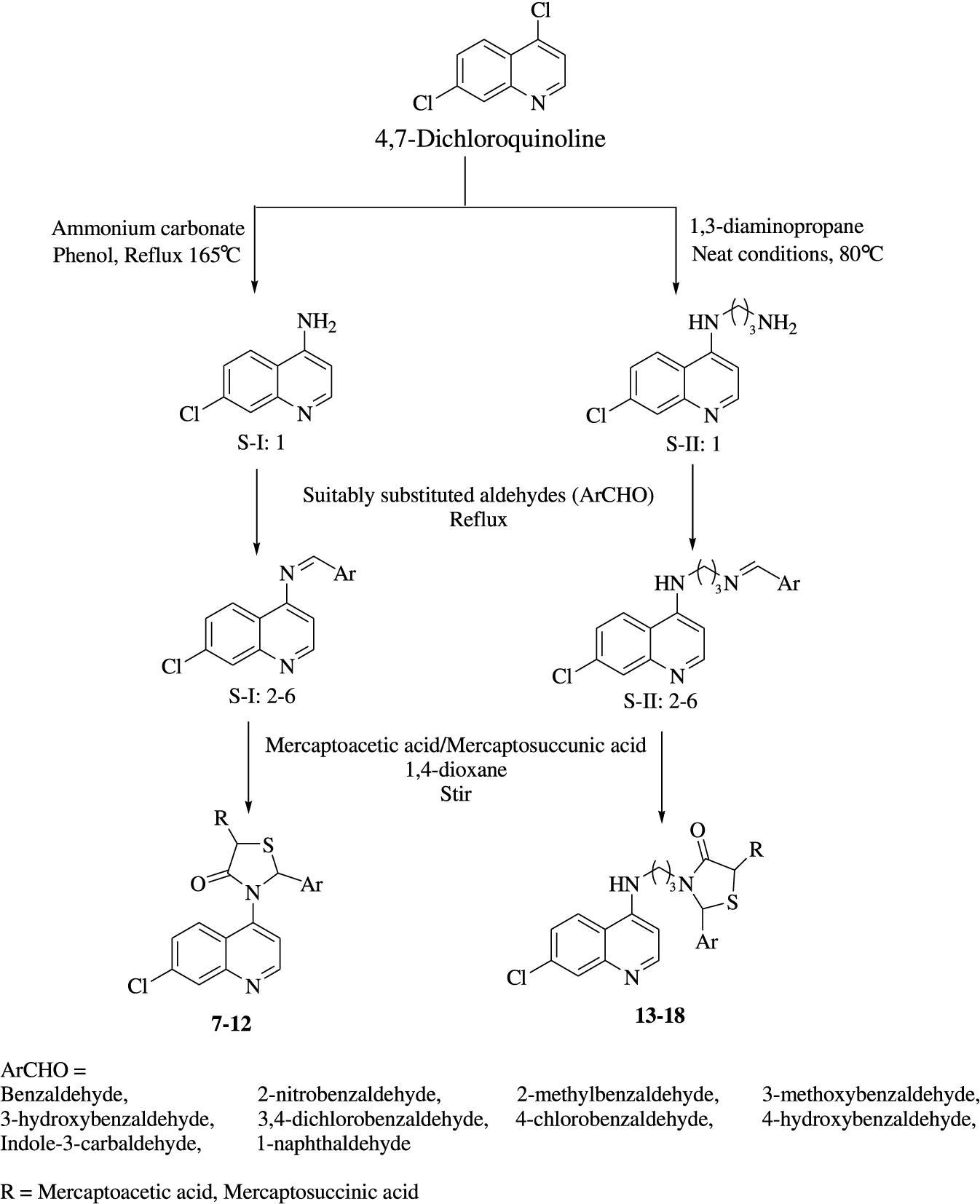
Syntheses of the 4-aminoquinoline-thiazolidinone hybrids, 7-18.

The title compounds, thiazolidine-4-one hybrids, were synthesized through a three-step reaction scheme, with bifurcation at step one. In the first step, 4-amino-7-chloroquinoline (SI:1) was synthesized using 4,7-dichloroquinoline (DCQ) and ammonium carbonate as the starting materials, while 4,7-diaminoquinoline (DCQ) and 1,3-diaminopropane under neat conditions yielded 4-diamino-7-chloroquinoline (SII:1). The second step involved converting both SI:1 and SII:1 to the corresponding Schiff bases, SI:2-6 and SII:2-6. The formed Schiff bases were heterocyclized to form the title compounds, 7-18, as given in Scheme 1. UV light and iodine vapors were used to visualize the reaction progress using pre-coated silica gel G plates.

#### 2.4.1 General procedure for syntheses of compounds of step 1

The compound SI:1 was synthesized by reaction of DCQ in phenol, followed by the addition of ammonium carbonate (Korotchenko et al., 2015). The compound SII:1 was synthesized by reacting DCQ and 1,3-diaminopropane in a molar ratio of 1:4, and characterized as already described in our previous report (Fatima et al., 2021).

##### 2.4.1.1 Characterization of the synthesized compound, SI:1

Light grey solid, yield 82%, m.p. 146 ˚C-148 C; IR (cm-^1^): 3343 (N–H stretch), 3031 (C-H sp^3^ stretch, aromatic), 1641 (N-H bend), 1508 (C=C stretch, aromatic), 1253 (C-N stretch), 761 (C-Cl stretch). MS (m/z) 179 (M+1)^+^.

#### 2.4.2 General procedure for syntheses of compounds of step 2

To a solution of the compound from step 1 in absolute ethanol, a catalytic amount of piperidine was introduced, followed by the addition of an excess (10x) of aldehyde. The reaction mixture was refluxed until the reaction was complete. It was subsequently cooled to room temperature and then poured onto crushed ice. To obtain the Schiff base, the isolated solid was washed and recrystallized (Fatima et al., 2021; Patel et al., 2012).

##### 2.4.2.1 Characterization of the synthesized compounds, SI:2-6 and SII:2-6

###### 2.4.2.1.1 (7-Chloro-quinolin-4-yl)-(1H-indol-3-ylmethylene)-amine **(SI:2)**

Reaction time: 13 h; Orange-colored sticky mass, yield 63%; IR (cm^-1^): 3565 (N–H stretch), 3100 (C-H sp^3^ stretch, aromatic), 1630 (N=C stretch), 1519 (N-H bend), 1440 (C=C stretch, aromatic), 1241 (C-N stretch), 757 (C-Cl stretch). MS (m/z) 306 (M+1)^+^.

###### 2.4.2.1.2 (7-Chloro-quinolin-4-yl)-naphthalen-1-ylmethylene-amine **(SI:3)**

Reaction time: 8 h; Dark brown liquid, yield 72%; IR (cm^-1^): 3051 (N–H stretch), 2726 (C-H sp^3^ stretch, aromatic), 1682 (N=C stretch), 1509 (N-H bend), 1458 (C=C stretch, aromatic), 1076 (C-N stretch), 767 (C-Cl stretch). MS (m/z) 317 (M+1)^+^.

###### 2.4.2.1.3 (7-Chloro-quinolin-4-yl)-(3,4-dichloro-benzylidene)-amine **(SI:4)**

Reaction time: 13 h; White-colored solid, yield 76%, m.p. 126 ˚C-128 C; IR (cm^-1^): 3445 (N–H stretch), 2977 (C-H sp^3^ stretch, aromatic), 1634 (N=C stretch), 1608 (C=C stretch, aromatic), 1506 (N-H bend), 1279 (C-N stretch), 812 (C-Cl stretch). MS (m/z) 335 (M+1)^+^.

###### 2.4.2.1.4 (7-Chloro-quinolin-4-yl)-(3-methoxy-benzylidene)-amine **(SI:5)**

Reaction time: 14 h; Cream-colored solid, yield 69%, m.p. 172 ˚C-174 C; IR (cm^-1^): 3611 (N–H stretch), 3013 (C-H sp^3^ stretch, aromatic), 1630 (N=C stretch), 1561 (C=C stretch, aromatic), 1461 (N-H bend), 1190 (C-N stretch), 1127 (C-O-C stretch), 824 (C-Cl stretch). MS (m/z) 297 (M+1)^+^.

###### 2.4.2.1.5 3-[(7-Chloro-quinolin-4-ylimino)-methyl]-phenol **(SI:6)**

Reaction time: 11 h; Brown solid, yield 74%, m.p. 164 ˚C-168 C; IR (cm^-1^): 3646 (OH stretch), 3565 (N–H stretch), 3195 (C-H sp^3^ stretch, aromatic), 1665 (N=C stretch), 1574 (C=C stretch, aromatic), 1490 (N-H bend), 1310 (C-N stretch), 1147 (C-O stretch), 780 (C-Cl stretch). MS (m/z) 283 (M+1)^+^.

###### 2.4.2.1.6 N-Benzylidene-N'-(7-chloro-quinolin-4-yl)-propane-1,3-diamine **(SII:2)**

Reaction time: 22 h; Golden brown liquid, yield 64%; IR (cm^-1^): 3566 (N–H stretch), 3064 (C-H sp^2^ stretch, aromatic), 1696 (N=C stretch), 1596 (C=C stretch), 1489 (N-H bend), 1455 (C-H sp^3^ bend, aliphatic), 1310 (C-N stretch), 743 (C-Cl stretch). MS (m/z) 324 (M+1)^+^.

###### 2.4.2.1.7 N-(4-chloro-benzylidene)-N'-(7-chloro-quinolin-4-yl)-propane-1,3-diamine **(SII:3)**

Reaction time: 24 h; Light off-white solid, yield 87%, m.p. 234 ˚C-238 C; IR (cm^-1^): 3512 (N–H stretch), 3028 (C-H sp^2^ stretch, aromatic), 1645 (N=C stretch), 1582 (C=C stretch), 1487 (N-H bend), 1452 (C-H sp^3^ bend, aliphatic), 1088 (C-N stretch), 822 (C-Cl stretch). MS (m/z) 358 (M)^+^.

###### 2.4.2.1.8 N-(7-chloro-quinolin-4-yl)-N'-(2-nitro-benzylidene)-propane-1,3-diamine **(SII:4)**

Reaction time: 23 h; Brown crystalline solid, yield 67%, m.p. 242 ˚C-244 C; IR (cm^-1^): 3457 (N–H stretch), 3070 (C-H sp^2^ stretch, aromatic), 1697 (N=C stretch), 1573 (C=C stretch), 1521 (N-H bend), 1443 (C-H sp^3^ bend, aliphatic), 1339 (C-N stretch), 732 (C-Cl stretch). MS (m/z) 369 (M+1)^+^.

###### 2.4.2.1.9 N-(7-chloro-quinolin-4-yl)-N'-(2-methyl-benzylidene)-propane-1,3-diamine **(SII:5)**

Reaction time: 25 h; White solid, yield 61%, m.p. 222 ˚C-224 C; IR (cm^-1^): 3421 (N–H stretch), 3053 (C-H sp^2^ stretch, aromatic), 1640 (N=C stretch), 1569 (C=C stretch), 1540 (N-H bend), 1452 (C-H sp^3^ bend, aliphatic), 1272 (C-N stretch), 744 (C-Cl stretch). MS (m/z) 338 (M+1)^+^.

###### 2.4.2.1.10 4-{[3-(7-Chloro-quinolin-4-ylamino)-propylimino]-methyl}-phenol **(SII:6)**

Reaction time: 28 h; Dark brown sticky mass, yield 58%; IR (cm^-1^): 3469 (N–H stretch), 3117 (C-H sp^2^ stretch, aromatic), 1664 (N=C stretch), 1574 (C=C stretch), 1509 (N-H bend), 1446 (C-H sp^3^ bend, aliphatic), 1210 (C-N stretch), 829 (C-Cl stretch). MS (m/z) 340 (M+1)^+^.

#### 2.4.3 General procedure for syntheses of compounds of step 3

A solution of the Schiff base (10 mmol) in 1,4-dioxane was reacted with thioacid (10 mmol) by stirring at room temperature. Upon completion of the reaction, the solvent was evaporated. The residue was washed with 4N Na_2_CO_3_ solution, followed by water washings. The obtained solid was filtered, washed with water until free of carbonate, subsequently washed with cold ethanol, and finally treated with ether. The compound was subsequently dried, recrystallized from methanol, and subjected to column chromatography to yield the title compounds (Fatima et al., 2025; Rahman et al., 2019)

##### 2.4.3.1 Characterization of the synthesized title compounds, 7-18

###### 2.4.3.1.1 3-(7-Chloro-quinolin-4-yl)-2-(1H-indol-3-yl)-thiazolidin-4-one (7)

Reaction time: 22 h; Cream-colored solid, yield 65%, m.p. 194-196 °C; IR (cm^-1^): 3032 (C-H sp^2^ stretch, aromatic), 1516 (C=C stretch), 1239 (C-N stretch), 755 (C-Cl stretch). PMR, δ, ppm: 3.34 (d, J = 15.01 Hz, 1H), 3.81 (d, J = 14.83 Hz, 1H), 7.21–7.23 (m, 2H), 7.24–7.26 (m, 2H), 7.49 (dd, J = 8.97, 2.20 Hz, 1H), 7.51 (dd, J = 6.97, 0.74 Hz, 1H), 8.28 (s, 1H), 8.46 (d, J = 4.76 Hz, 1H), 9.93 (d, J = 6.96 Hz, 1H). Found, %: C, 63.29; H, 3.74; N, 11.03. C_20_H_14_ClN_3_OS: Calculated, %: C, 63.24; H, 3.71; N, 11.06. Mass spectrum (ESI), m/z: [M+1]^+^ 380.

###### 2.4.3.1.2 [3-(7-Chloro-quinolin-4-yl)-2-(1H-indol-3-yl)-4-oxo-thiazolidin-5-yl]-acetic acid (8)

Reaction time: 21 h; Brown sticky mass, yield 68%; IR (cm^-1^): 2922 (C-H sp^2^ stretch, aromatic), 2312 (-OH stretch, carboxylic acid), 1702 (C=O stretch, carboxylic acid), 1416 (C=C stretch, aromatic), 1114 (C-N stretch), 1293 (C-O stretch, carboxylic acid), 889 (-OH oop, carboxylic acid), 745 (C-Cl stretch). ^1^H NMR, δ, ppm: 2.68 (dd, *J* = 15.66, 6.32 Hz, 1H), 3.46 (dd, *J* = 15.57, 6.22 Hz, 1H), 3.77 (t, *J* = 6.32, 6.32 Hz, 1H), 7.02 (d, *J* = 0.73 Hz, 1H), 7.12–7.23 (m, 2H), 7.27–7.45 (m, 2H), 7.50 (dd, *J* = 8.97, 2.20 Hz, 1H), 7.63 (dd, *J* = 6.96, 0.72 Hz, 1H), 7.65–8.08 (m, 1H), 8.10 (d, *J* = 2.01 Hz, 1H), 8.26 (d, *J* = 8.93 Hz, 1H), 8.38 (d, *J* = 4.76 Hz, 1H), 9.93 (d, *J* = 6.96 Hz, 1H). ^13^C NMR, δC, ppm: 36.32, 66.40, 111.92, 112.47, 118.21, 120.87, 121.74, 122.19, 124.16, 125.69, 126.08, 136.70, 137.10, 170.93. Found, %: C, 60.32; H, 3.71; N, 9.57. C_22_H_16_ClN_3_O_3_S: Calculated, %: C, 60.34; H, 3.68; N, 9.60. Mass spectrum (ESI), m/z: [M+1]^+^ 438.

###### 2.4.3.1.3 [3-(7-Chloro-quinolin-4-yl)-2-naphthalen-1-yl-4-oxo-thiazolidin-5-yl]-acetic acid (9)

Reaction time: 25 h; Cream-colored solid, yield 74%, m.p. 232-234 °C; IR (cm^-1^): 3051 (C-H sp^2^ stretch, aromatic), 2360 (-OH stretch, carboxylic acid), 1715 (C=O stretch, carboxylic acid), 1591 (C=C stretch, aromatic), 1167 (C-N stretch), 1215 (C-O stretch, carboxylic acid), 908 (-OH oop, carboxylic acid), 768 (C-Cl stretch). ^1^H NMR, δ, ppm: 2.82 (dd, *J* = 15.66, 6.32 Hz, 1H), 3.55 (dd, *J* = 15.57, 6.22 Hz, 1H), 3.80 (t, *J* = 6.32 Hz, 1H), 7.56 (s, 1H), 7.62 (d, *J* = 4.74 Hz, 1H), 7.64–7.70 (m, 5H), 7.75 (ddq, *J* = 6.20, 3.23, 1.48 Hz, 2H), 8.05 (d, *J* = 2.01 Hz, 1H), 8.07 (dd, *J* = 7.72, 1.60 Hz, 1H), 8.25 (d, *J* = 8.94 Hz, 1H), 9.13 (d, *J* = 4.76 Hz, 1H). ^13^C NMR, δC, ppm: 36.16, 40.13, 66.39, 115.21, 122.33, 125.43, 125.71, 126.39 - 126.89 (m, 4C), 127.59, 128.02, 128.76, 129.07, 133.35 (d, *J* = 5.2Hz), 134.58, 135.30, 136.83, 144.20, 156.93, 171.67, 172.21. Found, %: C, 64.23; H, 3.81; N, 6.28. C_24_H_17_ClN_2_O_3_S: Calculated, %: C, 64.21; H, 3.82; N, 6.24. Mass spectrum (ESI), m/z: [M+1]^+^ 448.

###### 2.4.3.1.4 [3-(7-Chloro-quinolin-4-yl)-2-(3,4-dichloro-phenyl)-4-oxo-thiazolidin-5-yl]-acetic acid (10)

Reaction time: 26 h; Brown semi-solid, yield 62%; IR (cm^-1^): 2929 (-OH stretch, carboxylic acid), 2852 (C-H sp^2^ stretch, aromatic), 1609 (C=C stretch, aromatic), 1367 (C-N stretch), 1257 (C-O stretch, carboxylic acid), 934 (-OH oop, carboxylic acid), 798 (C-Cl stretch). ^1^H NMR, δ, ppm: 2.58 (dd, *J* = 15.66, 6.32 Hz, 1H), 3.19 (dd, *J* = 15.57, 6.22 Hz, 1H), 3.82 (t, *J* = 6.23, 6.23 Hz, 1H), 6.81 (d, *J* = 1.00 Hz, 1H), 7.00–7.38 (m, 2H), 7.40–7.51 (m, 2H), 7.52 (dd, *J* = 2.13, 0.64 Hz, 1H), 7.76 (d, *J* = 2.01 Hz, 1H), 8.12 (d, *J* = 8.93 Hz, 1H), 8.30 (d, *J* = 4.76 Hz, 1H). ^13^C NMR, δC, ppm: 38.88, 39.51, 40.13, 102.76, 117.12, 123.84, 124.67, 127.32 (d, *J* = 1.9, 2C), 133.55, 149.44, 151.55, 151.73. Found, %: C, 51.36; H, 2.82; N, 5.95. C_20_H_13_ClN_2_O_3_S: Calculated, %: C, 51.35; H, 2.80; N, 5.99. Mass spectrum (ESI), m/z: [M+1]^+^ 467.

###### 2.4.3.1.5 [3-(7-Chloro-quinolin-4-yl)-2-(3-methoxy-phenyl)-4-oxo-thiazolidin-5-yl]-acetic acid (11)

Reaction time: 24 h; Off-white solid, yield 69%, m.p. 242-244 °C; IR (cm^-1^): 3502 (-OH stretch, carboxylic acid), 2952 (C-H sp^2^ stretch, aromatic), 1680 (C=O stretch, carboxylic acid), 1579 (C=C stretch, aromatic), 1338 (C-N stretch), 1273 (C-O-C stretch), 1223 (C-O stretch, carboxylic acid), 893 (-OH oop, carboxylic acid), 756 (C-Cl stretch). ^1^H NMR, δ, ppm: 2.49 (dd, *J* = 15.66, 6.32 Hz, 1H), 3.41 (dd, *J* = 15.57, 6.23 Hz, 1H), 6.55 (d, *J* = 0.73 Hz, 1H), 6.93 (dt, *J* = 7.69, 1.25, 1.25 Hz, 1H), 7.38 (t, *J* = 7.69, 7.69 Hz, 1H), 7.39–7.41 (m, 2H), 7.76 (dd, *J* = 8.97, 2.19 Hz, 1H), 8.17 (d, *J* = 2.02 Hz, 1H), 8.29 (d, *J* = 9.00 Hz, 1H), 8.74 (d, *J* = 4.76 Hz, 1H). ^13^C NMR, δC, ppm: 38.88, 40.13, 102.76, 109.00, 117.13, 121.87, 123.83, 127.33, 129.14, 130.85, 134.65, 135.82, 148.75, 149.44, 152.82, 155.13, 162.45. Found, %: C, 58.79; H, 4.03; N, 6.51. C_21_H_17_ClN_2_O_4_S: Calculated, %: C, 58.81; H, 4.00; N, 6.53. Mass spectrum (ESI), m/z: [M+1]^+^ 429.

###### 2.4.3.1.6 [3-(7-Chloro-quinolin-4-yl)-2-(3-hydroxy-phenyl)-4-oxo-thiazolidin-5-yl]-acetic acid (12)

Reaction time: 27 h; Light brown sticky mass, yield 71%; IR (cm-1): IR (cm^-1^): 3733 (-OH stretch, hydroxyl), 3097 (C-H sp^2^ stretch, aromatic), 2350 (-OH stretch, carboxylic acid), 1703 (C=O stretch, carboxylic acid), 1579 (C=C stretch, aromatic), 1400 (C-O stretch, hydroxyl), 1309 (C-O stretch, carboxylic acid), 1279 (C-N stretch), 941 (-OH oop, carboxylic acid), 779 (C-Cl stretch). ^1^H NMR, δ, ppm: 2.50 (dd, *J* = 15.66, 6.32 Hz, 1H), 2.82 (dd, *J* = 15.57, 6.22 Hz, 1H), 3.80 (t, *J* = 6.32, 6.32 Hz, 1H), 7.07 (d, *J* = 0.94 Hz, 1H), 7.23 (ddd, *J* = 8.23, 2.19, 1.27 Hz, 1H), 7.33 (td, *J* = 2.17, 2.16, 0.74 Hz, 1H), 7.35 (d, *J* = 4.73 Hz, 1H), 7.41–7.48 (m, 2H), 7.42 (d, *J* = 2.01 Hz, 1H), 9.90 (d, *J* = 8.93 Hz, 1H). ^13^C NMR, δC, ppm: 36.22, 40.13, 66.41, 114.72, 115.89, 120.06, 121.89, 125.90, 126.52, 129.65, 130.37, 137.72, 143.43, 157.44, 171.71. Found, %: C, 57.92; H, 3.61; N, 6.77. C_20_H_15_ClN_2_O_4_S: Calculated, %: C, 57.90; H, 3.64; N, 6.75. Mass spectrum (ESI), m/z: [M+1]^+^ 415.

###### 2.4.3.1.7 3-[3-(7-Chloro-quinolin-4-ylamino)-propyl]-2-phenyl-thiazolidin-4-one (13)

Reaction time: 28 h; Pale-yellow sticky mass, yield 65%; IR (cm^-1^): 3648 (N–H stretch), 2957 (sp^2^ stretch, aromatic), 1725-1705 (C=O stretch, ketonic), 1595 and 1452 (C=C stretch, ring), 1557 (N-H bend, amine), 1361 (-CH_2_- bend, aliphatic), 1252 (C-N stretch, amine), 1211 (C-O stretch, carboxylic acid), 1116 (C=O bend, ketonic) 1080 (C-Cl stretch), 869 (=C-H oop, aromatic). ^1^H NMR, δ, ppm: 2.50 (dt, *J* = 13.18, 5.44 Hz, 1H), 3.62 (d, *J* = 15.01 Hz, 1H), 3.70 (d, *J* = 14.83 Hz, 1H), 5.83 (d, *J* = 0.84 Hz, 1H), 6.46 (d, *J* = 4.22 Hz, 1H), 7.24 (dd, *J* = 8.79, 2.20 Hz, 1H), 7.49–7.53 (m, 3H), 7.63 (d, *J* = 8.61 Hz, 1H), 7.85 (d, *J* = 2.07 Hz, 1H), 7.93 (dddd, *J* = 7.50, 4.33, 2.21, 1.58 Hz, 2H), 7.96 (t, *J* = 5.13 Hz, 1H). ^13^C NMR, δC, ppm: 25.11, 34.25, 40.34, 41.11, 62.25, 115.24, 124.80, 125.40, 127.50 (d, *J* = 17.7 Hz, 2C), 128.13, 129.11, 129.16 (d, *J* = 6.4 Hz, 2C), 129.49, 134.58, 140.15, 146.41, 149.67, 151.22, 170.86. Anal. Calcd. for: C_21_H_20_ClN_3_OS: C, 63.39; H, 5.07; N, 10.56. Found: C, 63.42; H, 5.05; N, 10.59. MS (m/z) 398 (M+1)+.

###### 2.4.3.1.8 {3-[3-(7-Chloro-quinolin-4-ylamino)-propyl]-4-oxo-2-phenyl-thiazolidin-5-yl}-acetic acid (14)

Reaction time: 29 h; Cream-coloured solid, yield 77%, m.p. 130-132 °C; IR (cm^-1^): 3740 (N–H stretch), 2919 (sp^2^ stretch, aromatic), 2853 (O-H stretch, carboxylic acid), 1681 (C=O stretch, ketonic), 1713 (C=O stretch, carboxylic acid), 1608 and 1411 (C=C stretch, ring), 1525 (N-H bend, amine), 1278 (-CH_2_- bend, aliphatic), 1171 (C-O stretch, carboxylic acid), 1069 (C=O bend, ketonic) 1023 (C-Cl stretch), 917 (C-N stretch, amine), 689 (=C-H oop, aromatic). ^1^H NMR, δ, ppm: 2.49 (pd, *J* = 5.62, 1.92 Hz, 2H), 2.58–2.63 (m, 2H), 2.78–2.82 (m, 1H), 3.53–3.57 (m, 3H), 7.47–7.51 (m, 3H), 7.60 (d, *J* = 8.60 Hz, 1H), 7.92 (d, *J* = 2.07 Hz, 1H), 7.95 (dddd, *J* = 7.42, 4.33, 2.21, 1.58 Hz, 2H). ^13^C NMR, δC, ppm: 30.69, 35.63, 39.48 (d, *J* = 6.2 Hz, 2C), 128.56 (d, *J* = 17.6 Hz, 2C), 129.25, 130.73, 132.86, 167.31, 171.80, 173.53. Anal. Calcd. for: C_23_H_22_ClN_3_O_3_S: C, 60.59; H, 4.86; N, 9.22. Found: C, 60.62; H, 4.81; N, 9.25. MS (m/z) 456 (M+1)+.

###### 2.4.3.1.9 {2-(4-Chloro-phenyl)-3-[3-(7-chloro-quinolin-4-ylamino)-propyl]-4-oxo-thiazolidin-5-yl}-acetic acid (15)

Reaction time: 32 h; Dark off-white solid, yield 85%, m.p. 224-226 °C; IR (cm^-1^): 3742 (N–H stretch), 2920 (sp^2^ stretch, aromatic), 2848 (O-H stretch, carboxylic acid), 1710 (C=O stretch, ketonic), 1684 (C=O stretch, carboxylic acid), 1565 and 1374 (C=C stretch, ring), 1473 (N-H bend, amine), 1274 (-CH_2_- bend, aliphatic), 1140 (C-N stretch, amine), 1196 (C-O stretch, carboxylic acid), 1080 (C=O bend, ketonic) 1007 (C-Cl stretch), 807 (=C-H oop, aromatic). ^1^H NMR, δ, ppm: 2.08 (pd, *J* = 5.63, 1.92 Hz, 2H), 2.50–2.52 (m, 2H), 3.34 (dd, *J* = 15.66, 6.50 Hz, 1H), 7.66 (d, *J* = 4.21 Hz, 1H), 7.68 (s, 4H), 7.91 (d, *J* = 8.53 Hz, 1H), 10.00 (t, *J* = 5.13 Hz, 1H). ^13^C NMR, δC, ppm: 30.65, 39.49 (d, *J* = 6.7 Hz, 2C), 129.34, 131.15, 134.81, 139.34, 192.11. Anal. Calcd. for: C_23_H_21_C_l2_N_3_O_3_S: C, 56.33; H, 4.32; N, 8.57. Found: C, 56.29; H, 4.30; N, 8.54. MS (m/z) 490 (M+1)+.

###### 2.4.3.1.10 3-[3-(7-Chloro-quinolin-4-ylamino)-propyl]-2-(2-nitro-phenyl)-thiazolidin-4-one (16)

Reaction time: 34 h; Golden brown sticky mass, yield 87%; IR (cm^-1^): 3510 (N–H stretch), 3004 (sp^2^ stretch, aromatic), 1707 (C=O stretch, ketonic), 1531 and 1357 (C=C stretch, ring), 1424 (N-H bend, amine), 1221 (C-N stretch, amine), 1357 (C-O stretch, carboxylic acid), 1092 (C=O bend, ketonic) 902 (C-Cl stretch), 788 (=C-H oop, aromatic). ^1^H NMR, δ, ppm: 1.14 (pd, *J* = 5.56, 2.02 Hz, 2H), 3.36–3.44 (m, 2H), 3.72 (d, *J* = 15.01 Hz, 1H), 3.73 (d, *J* = 14.83 Hz, 1H), 5.09 (d, *J* = 0.71 Hz, 1H), 5.27 (d, *J* = 4.22 Hz, 1H), 5.33 (dd, *J* = 8.79, 2.20 Hz, 1H), 5.65 (dd, *J* = 9.00, 1.27 Hz, 1H), 5.88–5.91 (m, 2H), 7.74 (d, *J* = 2.08 Hz, 1H). ^13^C NMR, δC, ppm: 25.31, 34.31, 39.79, 41.01, 58.03, 116.00, 121.54, 124.60, 125.50, 129.49, 129.84, 129.99, 134.96, 141.50, 146.75, 147.60, 149.12, 153.76, 171.98. Anal. Calcd. for: C_21_H_19_ClN_4_O_3_S: C, 56.95; H, 4.32; N, 12.65. Found: C, 56.99; H, 4.29; N, 12.61. MS (m/z) 443 (M+1)+.

###### 2.4.3.1.11 3-[3-(7-Chloro-quinolin-4-ylamino)-propyl]-2-o-tolyl-thiazolidin-4-one (17)

Reaction time: 24 h; White solid, yield 76%, m.p. 208-210 °C; IR (cm^-1^): 3619 (N–H stretch), 3021 (sp^2^ stretch, aromatic), 1743 (C=O stretch, carboxylic acid), 1707 (C=O stretch, ketonic), 1610 and 1457 (C=C stretch, ring), 1546 (N-H bend, amine), 1347 (-CH_2_- bend, aliphatic), 1309 (C-N stretch, amine), 1347 (C=O bend, ketonic), 1025 (C-Cl stretch), 737 (=C-H oop, aromatic). ^1^H NMR, δ, ppm: 2.08 (pd, *J* = 5.56, 2.02 Hz, 2H), 2.22 (d, *J* = 0.60 Hz, 3H), 2.49 (dt, *J* = 13.18, 5.44 Hz, 1H), 3.24–3.26 (m, 2H), 3.56 (d, *J* = 15.01 Hz, 1H), 3.68 (d, *J* = 14.83 Hz, 1H), 6.01 (d, *J* = 0.62 Hz, 1H), 6.46 (d, *J* = 4.22 Hz, 1H), 7.13–7.17 (m, 2H), 7.19 (ddd, *J* = 8.17, 5.76, 1.88 Hz, 2H), 7.46 (ddd, *J* = 7.88, 6.86, 2.03 Hz, 1H), 7.80 (d, *J* = 8.61 Hz, 1H), 7.81 (d, *J* = 2.08 Hz, 1H), 8.38 (d, *J* = 4.40 Hz, 1H), 8.40 (t, *J* = 5.13 Hz, 1H). ^13^C NMR, δC, ppm: 18.36, 25.14, 30.67, 39.49, 98.67, 117.21, 124.21, 126.50 (d, *J* = 8.1 Hz, 2C), 128.17, 133.90, 147.79, 150.45, 150.83, 170.49. Anal. Calcd. for: C_24_H_24_ClN_3_O_3_S: C, 61.33; H, 5.15; N, 8.94. Found: C, 61.34; H, 5.17; N, 8.90. MS (m/z) 412 (M+1)+.

###### 2.4.3.1.12 [3-[3-(7-Chloro-quinolin-4-ylamino)-propyl]-2-(4-hydroxy-phenyl)-4-oxo-thiazolidin-5-yl]-acetic acid (18)

Reaction time: 29 h; Dark brown solid, yield 73%, m.p. 236-238 °C; IR (cm^-1^): 3503 (N–H stretch), 3444 (O-H stretch, carboxylic acid), 2915 (sp^2^ stretch, aromatic), 1714 (C=O stretch, ketonic), 1680 (C=O stretch, carboxylic acid), 1610 and 1473 (C=C stretch, ring), 1511 (N-H bend, amine), 1396 (-CH_2_- bend, aliphatic), 1338 (C-O stretch, carboxylic acid), 1317 (C=O bend, ketonic), 1259 (C-N stretch, amine), 1015 (C-Cl stretch), 900 (=C-H oop, aromatic). ^1^H NMR, δ, ppm: 1.74 (pd, J = 5.62, 1.92 Hz, 2H), 2.71–2.78 (m, 2H), 3.39–3.42 (m, 1H), 3.58 (t, J = 6.50 Hz, 1H), 5.36 (s, 1H), 6.73 (s, 1H), 6.74–6.76 (m, 2H), 6.90–6.94 (m, 2H), 7.22 (dd, J = 8.79, 2.20 Hz, 1H), 7.76 (d, J = 8.61 Hz, 1H), 7.77 (d, J = 2.08 Hz, 1H), 9.77 (t, J = 5.13 Hz, 1H). ^13^C NMR, δC, ppm: 25.20, 36.34, 39.51, 42.94 (d, *J* = 6.2 Hz, 2C), 51.33, 67.10, 116.22, 128.52, 129.18, 132.19, 145.46, 157.4, 163.39, 172.31, 191.04. Anal. Calcd. for: C_23_H_22_ClN_3_O_4_S: C, 58.53; H, 4.70; N, 8.90. Found: C, 58.49; H, 4.68; N, 8.87. MS (m/z) 472 (M+1)+.

## 3 Biological activity

The title compounds were tested for anti-malarial activity using the *Pf*-DHFR *in-vitro* enzyme inhibition assay, as well as the *Plasmodium berghei* murine model.

### 3.1 *In vitro* study: *Pf*-DHFR inhibition assay

The enzyme *Pf*-DHFR, dihydrofolic acid, and NADPH were obtained from Merck, Germany. The enzyme inhibitory assay was conducted as described in the literature (Bilsland et al., 2018). The assay mixture comprised a sodium acetate buffer pH 6.0 (1.5 M), NADPH (50 *µ*M), 0.6 M KCl, dimethylsulphoxide (20 *µ*L) at a concentration of 10^−11^ to 10^−5^ M, with 0.02 units of *Pf*-DHFR, totaling a final volume of 2 mL. Following the addition of the enzyme, the mixture was incubated at 25 °C for 5 min, and 25 *µ*M dihydrofolic acid was added to initiate the reaction. The change in absorbance (ΔA per minute) was recorded at 340 nm. The activity was monitored for 10 min under the above conditions. The results are presented (in triplicate) as percentage suppression of enzymatic activity. The percent inhibition was calculated using Equation 1, followed by calculation of the IC_*50*_ values.

$$\%\;Inhibition=\left(1-\frac{\frac{\Delta A}{\min\left\{test\right\}}}{\frac{\Delta A}{\min\left\{DMSO\right\}}}\right)*100$$ (1)

### 3.2 *In vivo* study: primary schizonticidal screening (Peter’s four-day suppressive test)

For the study, 4 to 6 weeks-old albino male mice weighing 25–30 g were used. The animals were obtained from the Animal House Facility. All the animals were handled in accordance with the guidelines specified by the Institutional Animal Ethics Committee (IAEC). The biological activities received clearance from the IAEC under protocol number BBDNIIT/IAEC/2019/12. The animals were divided into groups with n = 5. The treatments, comprising the standard, the vehicle, and the test compounds, were administered orally at a dosage of 1 mL/100 g body weight. Chloroquine (CQ) and Artesunate (ART) were the standard drugs used.

The study protocol adopted in the study is as described in the previous literature (Mehrotra et al., 2019). The malaria parasite strain, *P. berghei,* was obtained from the National Institute of Malaria Research, New Delhi, India. The parasites were sustained through re-infestation, followed by inoculum preparation by diluting blood with RPMI-1640 culture medium to attain a concentration of 1 × 10^^7^ infected erythrocytes/mL of the blood. The mice were infected intraperitoneally with the inoculum. The positive control, negative control, and the test compounds were administered orally for four consecutive days. On the fifth day, following 24 h post-administration of the final dose, thin smears were prepared from the tail vein blood of each animal. The blood films were subsequently dried and fixed using methanol. They were stained with 10% Giemsa stain in phosphate buffer of pH 7.2 and analyzed under a microscope to quantify parasites per 10,000 RBCs/mm^3^ employing Neubauer’s Chamber. The percentage parasitaemia (% P) and percentage parasitaemia inhibition (% PI) were calculated using Equations 2 and 3, and the survival time in days was recorded, and the mean was calculated over a period of 29 days employing Equation 4.

$$\%\;P=\frac{Number\;of\;parasitized\;RBC}{Total\;number\;of\;RBC\;counted}\;*\;100$$ (2)

$$\%\;PI=\frac{\%\;P\;in\;negative\;control-\%\;P\;in\;treatment\;group}{\%\;P\;in\;negative\;control}\;*100$$ (3)

$$MST=\frac{Sum\;of\;survival\;time\;of\;all\;mice\;in\;agroup\;\left(days\right)}{Total\;number\;of\;mice\;in\;that\;group}$$ (4)

### 3.3 Statistical analysis

The experimental results are reported as mean ± SD for *in-vitro* studies and mean ± SEM for *in-vivo* studies of three independent experiments. The % P was analyzed using one-way ANOVA, and the % PI and MST were analyzed using two-way ANOVA. Multiple comparisons of row statistics were performed with Dunnett’s test and Brown–Forsythe and Welch ANOVA tests, with statistical significance set at P < 0.05. The IC_*50*_ values were derived from log-dose response curves normalized to control. The statistical analyses were carried out using GraphPad Prism 9.0.

### 3.4 Acute toxicity study

The synthesized compounds were tested for acute toxicity (LD50) using OECD guideline 423 (OECD, 2001). After 4 hours of fasting, the compounds were given orally at 5, 50, 300, and 2000 mg/kg body weight. Food, but not water, was withheld for 2 hours after dosing. D0, D1, D7, and D14 were research days for the mouse body weight. The animals were examined for 4 hours after dose administration and then up to 14 days for mortality, including toxicity indicators like behavioral changes, skin-fur abnormalities, tremors, or death.

## 4 Results and discussion

### 4.1 Computational study: molecular docking

The presence of a high-quality protein crystal structure and the implementation of an optimal docking methodology are the two crucial requirements for the efficacy of docking simulations. Therefore, before performing simulations for the designed molecules, it was decided to optimize the target protein crystal structures and validate the docking procedure.

**FIGURE 2:**
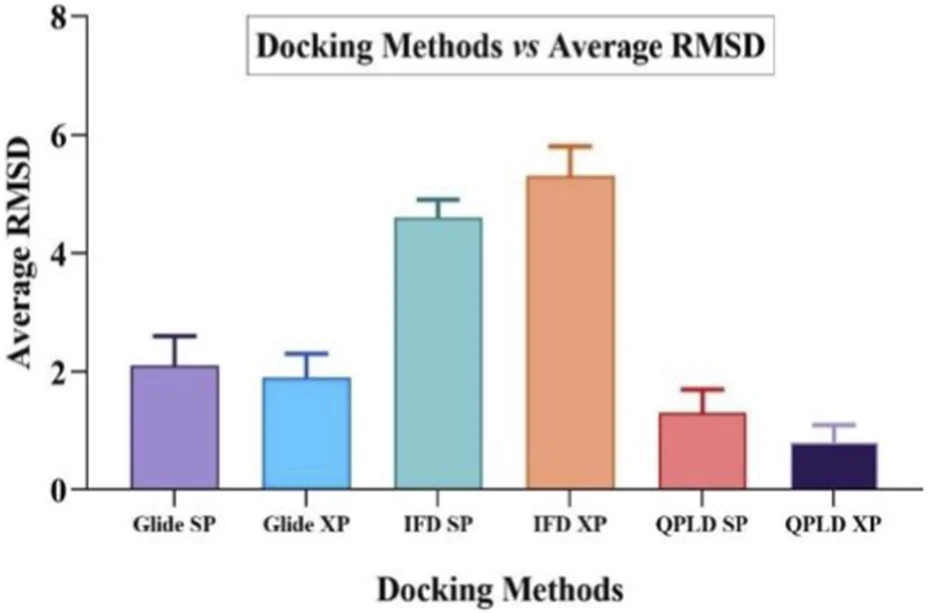
Graph representing the docking validation.

When compared to the other docking protocols used, the study’s results indicated that the QPLD-XP approach was the most successful (Figure 2).

**FIGURE 3:**
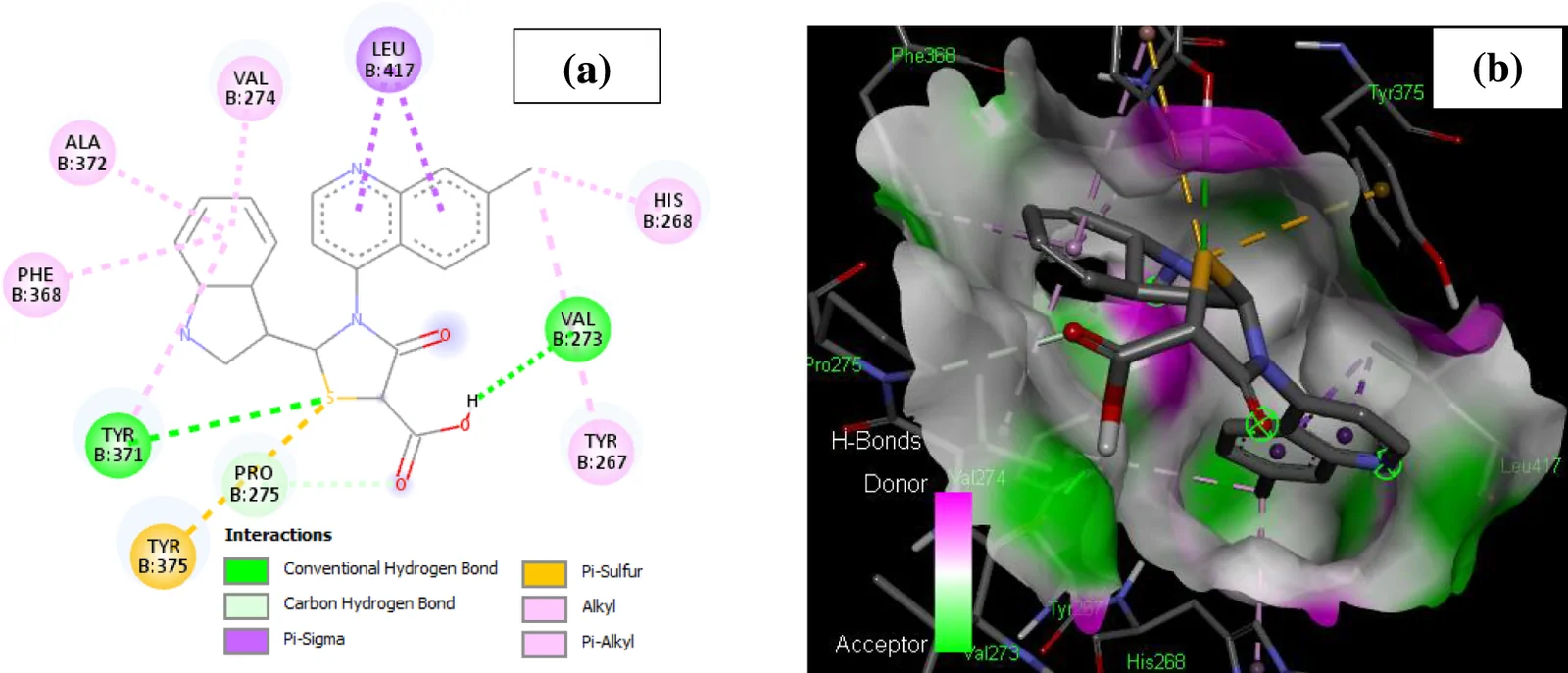
Interaction diagrams **(a)** 2D and **(b)** 3D depicting the ligand, 8, at the binding site of the *plasmodial* protein, 3UM5.

The molecules were chosen using the molecular docking studies according to the target affinity and binding properties against the biological targets. From the study, it could be inferred that the most promising compound 8, with XP GScore 9.615 and shape similarity 0.317, displayed interaction of the amino acid residues *Tyr 267*, *His 268*, and *Leu 417*, with the 7-Cl, 4-AQ, along with *Val 273*, *Pro 275*, *Tyr 371* and *Tyr 375* interacting with the 4-THZ, at the binding site of the *Pf*-DHFR target protein (PDB ID 3UM5). Conventional hydrogen bonds, carbon-hydrogen bonds, *pi*-sigma, *pi*-sulfur, alkyl, and *pi*-alkyl interactions are among the interactions (Figure 3). Meanwhile, compound 15 (XP GScore -9.346) displayed alkyl/*pi*-alkyl interactions of its quinoline ring with the amino acid residues *Tyr 379, Leu 417, Met 420,* and *Pro 423.* The *Pf*-DHFR inhibition of the molecules can be attributed to the strong interactions with the amino acid residues, including *Val 273, Tyr 371,* and *Tyr 375,* with the receptor site through conventional *H*-bond, *pi-pi* T-shaped, and *pi*-sigma interactions. In addition, the other target *plasmodial* enzymes against which the designed molecules displayed good binding affinities include PDB IDs 1LF2, BWK, and 3LT0. The XP GScore for the designed molecules and their shape similarity is given in Table 1.

**TABLE 1:** Docking scores of the designed hybrids, 7-18.

Comp. code	PDB ID (protein target)	XP GScore (Kcal/mol)	Shape similarity
7	3LT0	−6.834	0.378
8	3UM5	−9.615	0.317
9	3UM5	−9.196	0.350
10	3LT0	−8.693	0.340
11	3BWK	−9.095	0.337
12	3UM5	−6.592	0.366
13	3LT0	−7.009	0.560
14	3UM5	−9.380	0.431
15	3UM5	−9.346	0.429
16	3BWK	−6.581	0.564
17	1LF2	−7.226	0.476
18	3BWK	−9.156	0.382

### 4.2 Computational study: molecular dynamics

The MD simulation study gave insight into structural and conformational changes that occurred during the controlled simulated physiological environment. The effect of various factors on the binding interactions between the receptor and the ligand was understood, and the following parameters were analyzed.

#### 4.2.1 The root mean square deviation (RMSD)

**FIGURE 4:**
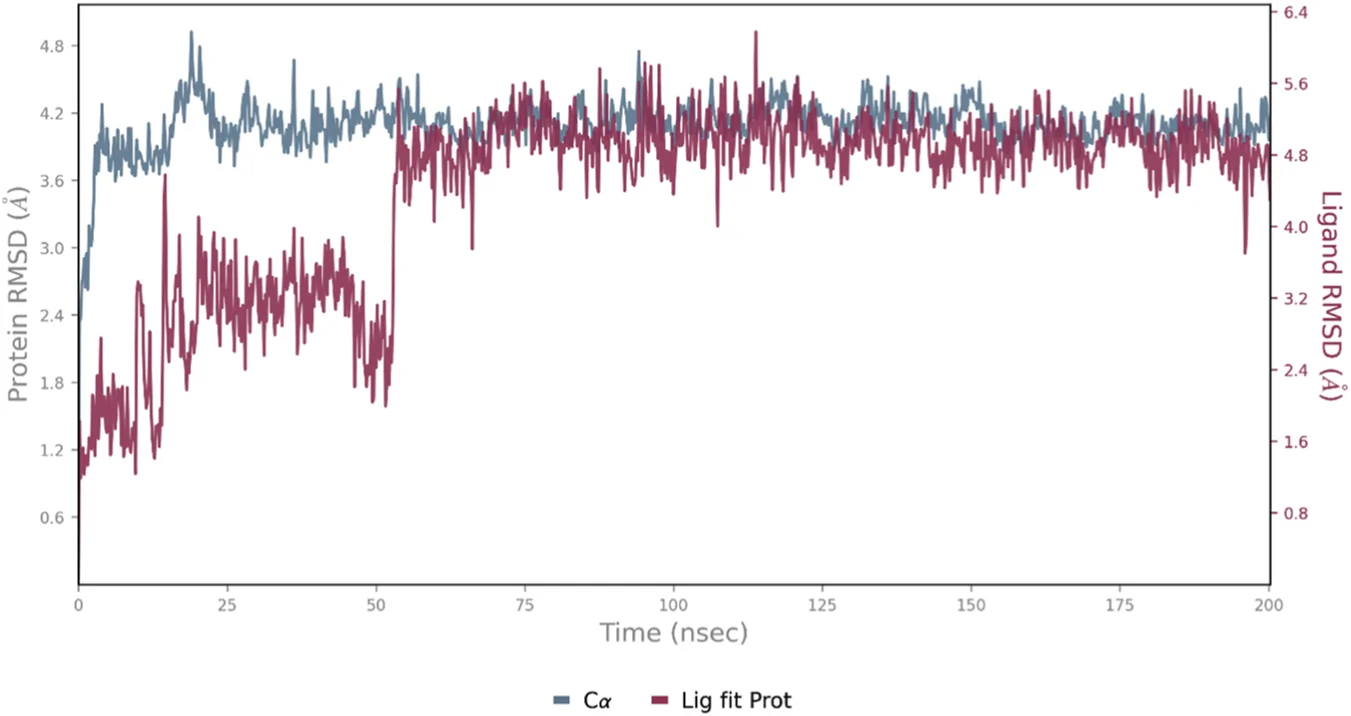
Molecular dynamics simulation depicting the Root Mean Square Deviation.

The RMSD of the protein-ligand complex is as shown in the figure below. The protein backbone and ligand in the protein complex RMSD are depicted as blue and maroon, for Cα and Lig fit Prot, respectively (Figure 4). Initially, for 25 ns, protein RMSD showed minor fluctuations from 2.4 to 4.2 Å, which indicates the initial equilibration. After 25 ns, the protein RMSD was found to be quite stable and had RMSD value of 4.2 Å throughout the complete simulation study.

The ligand was also subjected to stabilization into the binding pocket; hence, for the initial 20 ns, the ligand showed slight fluctuations of 1 to 3 Å. After 20 ns, the ligand was stable until 50 ns, and had RMSD value of approximately 2.6 Å, indicating a good interaction. After 50 ns, the ligand showed stability till the 200 ns and had an RMSD value of 4 Å. The RMSD of the ligand was calculated following its alignment within the binding site of the protein, revealing greater fluctuations compared to the protein. These fluctuations could be because the ligand RMSD reflected both internal flexibility and positional adjustments inside the binding pocket. The RMSD analysis revealed that the formed complex was stable and the fluctuations were associated with only equilibration and conformational changes.

#### 4.2.2 The ligand root mean square fluctuation (RMSF)

**FIGURE 5:**
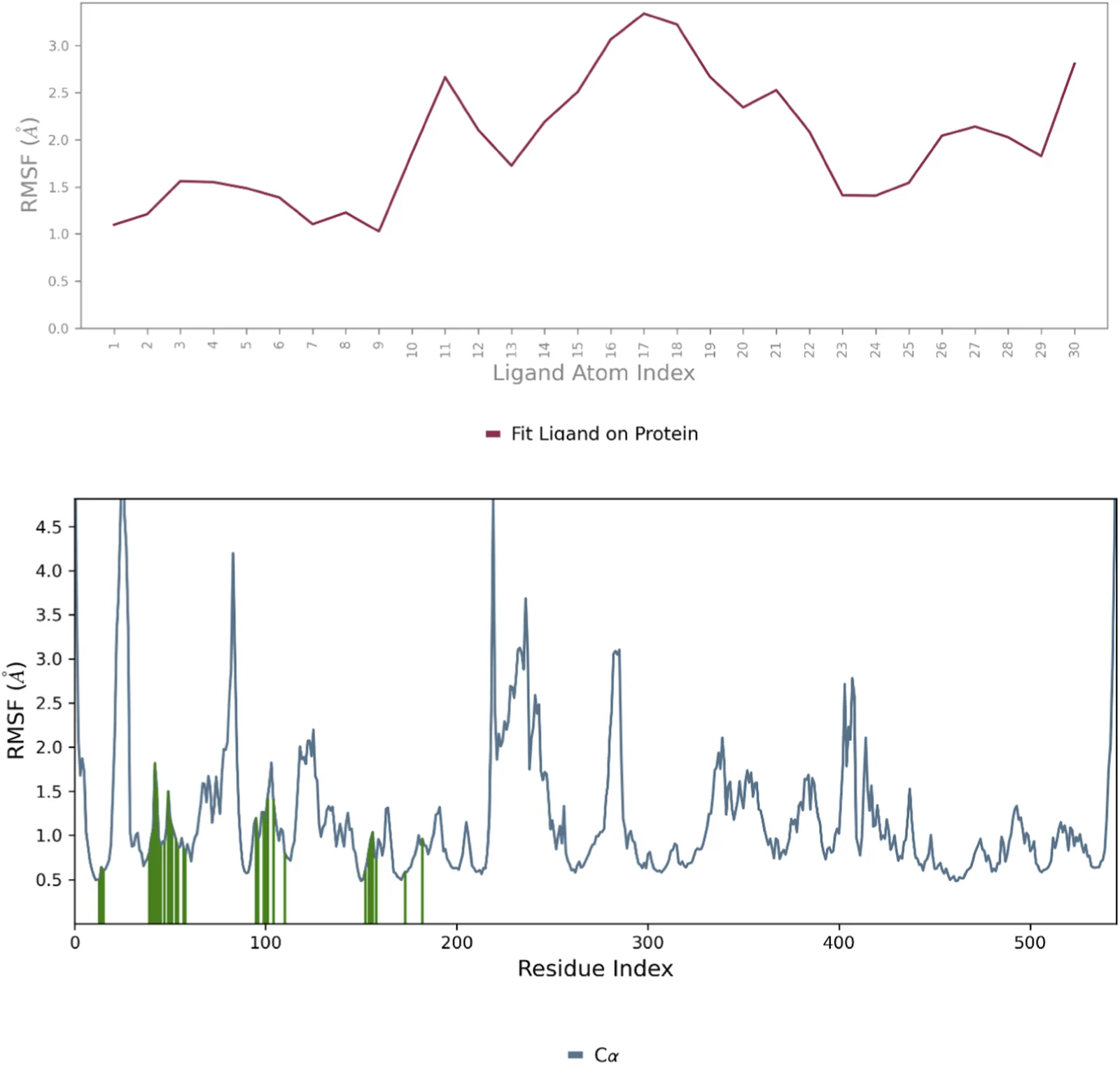
Molecular dynamics simulation depicting the root mean square fluctuation.

The RMSF was performed to determine the flexibility of the amino acid residues. The green bars indicate the residues involved in the ligand interactions during the simulation study. The ligand atoms of the complex showed RMSF in the range of 1–3.5 Å (Figure 5).

The slight high RMSF was observed in ligand atoms 13-20, which belong to the indole ring attached with a single bond to the scaffold. Probably the ring has undergone conformational adjustment; hence, the RMSF was observed as 2-3.5 Å. Similar was found with the chlorine atom number 30, having an RMSF value of 3 Å. The protein residues of the complex showed an RMSF of 0.5–4.75 Å. The amino acid residues of the terminal region showed the maximum RMSF values of 3.5–4.75 Å, as these were free from intramolecular interaction and also more accessible to the solvent. Residues numbered 210 to 240 again had the same value of RMSF due to conformational adjustments. The rest of the residues showed an average RMSF of approximately 2 Å, which is considered good. These small fluctuations indicate the stability of the protein and ligand as well during the MD simulation study.

#### 4.2.3 Protein-ligand (PL) interaction

**FIGURE 6:**
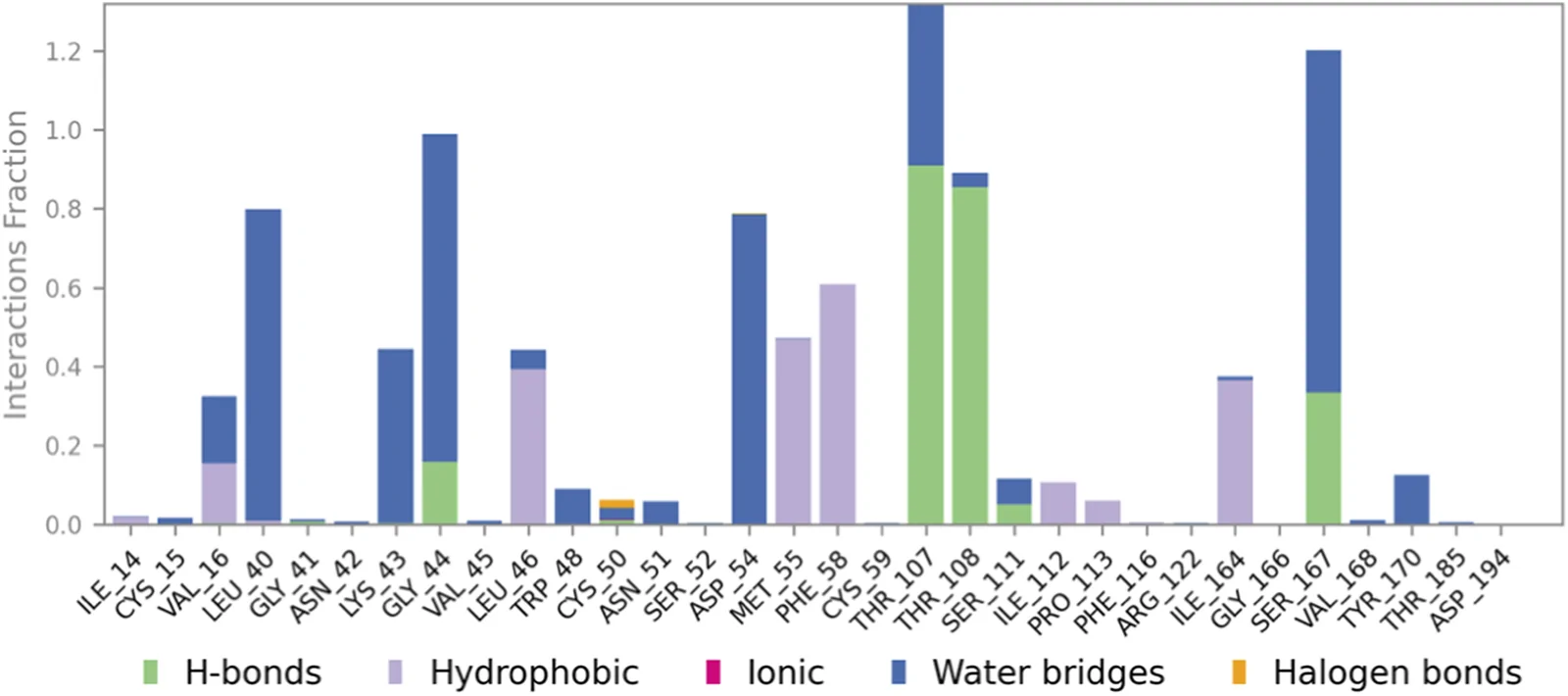
Molecular dynamics simulation depicting the protein-ligand interaction.

PL interaction with the ligand was monitored throughout the study. The PL histogram (Figure 6) revealed that the five amino acids, *Gly44, Thr107, Thr108, Ser111,* and *Ser167*, had H-bond contacts. The hydrophobic interaction was observed by *Val16, Leu46, Met55, Phe58, Ile112, Pro113* and *Ile164* residues. Water bridges were also observed by many residues as *Val16, Leu40, Lys43, Gly44, Leu46, Trp48, Cys50, Asn51, Asp54, Thr107, Thr108, Ser111, Ser167* and *Tyr170*. The halogen atom (Cl) of the ligands showed interaction with *Cys50*. Conclusively, the complex was stabilized mainly by hydrogen and hydrophobic interactions, complemented by the halogen bond and water bridges.

**FIGURE 7:**
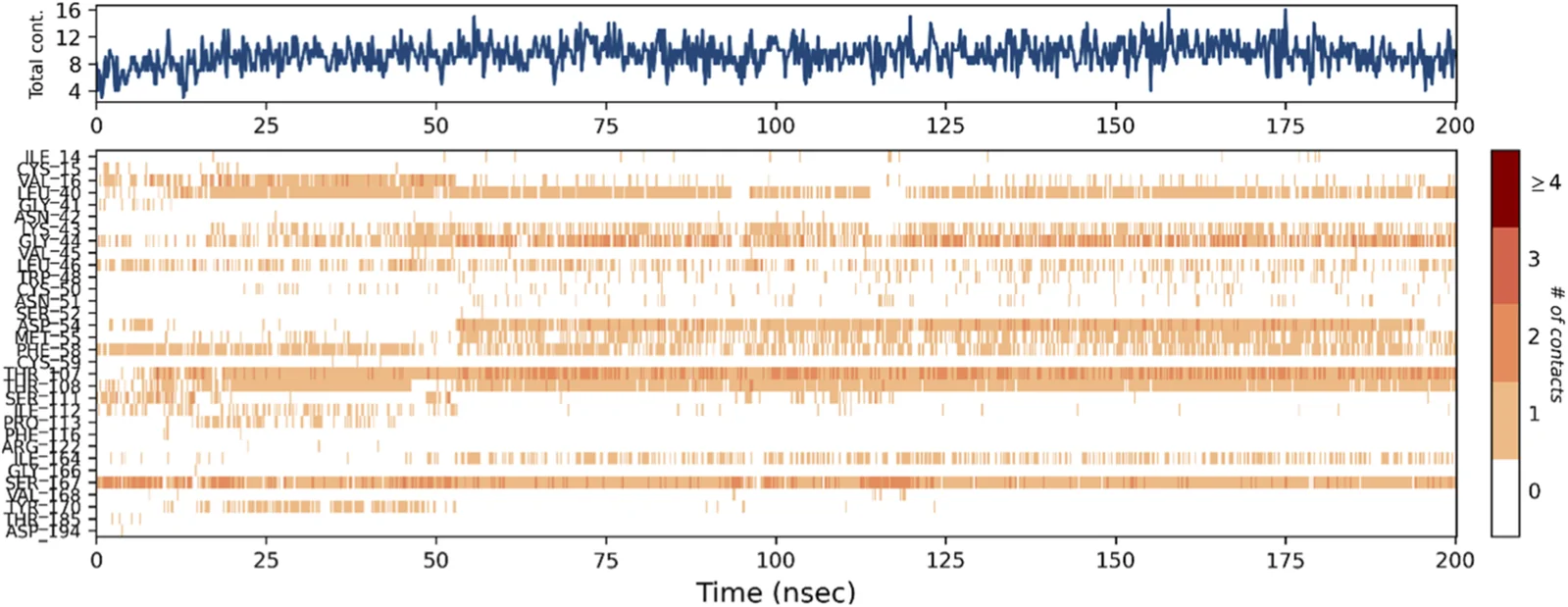
Timeline depicting the protein-ligand interaction presenting stability confirmation.

The timeline (Figure 7) shows protein-ligand interactions and contacts, including H-bonds, hydrophobic, ionic, water bridges, and halogen bonds. The image shows the total number of protein-ligand contacts in the upper panel and the amino acid residue interaction and dominance in the lower panel. The color intensity shows the number of interactions between the amino acid residue and the ligand with respect to time. The graph revealed that 6-7 interactions were more stable than the other interactions. The amino acid residues *Thr107, Thr108,* and *Ser167* were found to interact the most throughout the 200 ns. Other interacting amino acid residues were *Leu40, Lys43, Gly44, Leu46,* and *Phe58*. The multiple interacting residues in the simulation study indicated the ligand’s stability at the binding site. Also, the ligand-protein interaction corroborated well with the RMSD and RMSF analysis to confirm that the complex was stable.

### 4.3 ADMET prediction

The *in silico* ADME properties for all the selected molecules were carried out using the StarDrop software from Optibrium, Cambridge, United Kingdom. None of the molecules were found to violate Lipinski’s rule of five. The compounds also exhibited good *i.v.* non-CNS and *oral* non-CNS activity, therefore presenting commendable bioavailability paradigms. The predicted hEGR pIC_*50*_ values for the compounds were found to fall within the limits, suggesting the molecules are devoid of any cardiac toxicity. Also, the compounds were predicted to display good human intestinal absorption. In addition, the compounds were predicted as substrates to P-glycoprotein and exhibited metabolism and affinity to various cytochrome models (2C9 *p*K*i* and 2D6 affinity. The complete prediction data are given in Table 2. The *in silico* toxicity assessment for all the designed molecules was carried out using the OSIRIS Property Explorer (Dong et al., 2018; Klebe, 2000) and LAZAR (Cheng et al., 2012; Yang, 2010) freeware programs available online, and is given in Table 3. It was found that the molecules, particularly those with higher binding affinity, were completely devoid of any potential toxicity risks such as acute toxicity, carcinogenicity-tumorigenicity, mutagenicity, reproductive toxicity, and irritancy.

**TABLE 2:** The ADME prediction data of the designed hybrids, 7-18.

Comp. code	*i. v.* Non-CNS	Oral non-CNS	log S	log S @ pH 7.4	2C9 pKi	hERG pIC_*50*_	HIA cat.	P-gp cat.	2D6 Affinity category	PPB9 category	Lipinski’s rule of five
7	0.06642 ± 0.0715	0.03051 ± 0.0540	−0.2201 ± 1.033	0.5372 ± 0.621	5.87 ± 0.6013	5.156 ± 0.5973	+	Yes	High	High	0.9905 ± 0.0684
8	0.0889 ± 0.0980	0.0441 ± 0.0793	−0.3608 ± 1.033	1.32 Inf	5.826 ± 0.6013	4.128 ± 0.6834	+	Yes	High	High	0.998 ± 0.0319
9	0.05099 ± 0.0234	0.01959 ± 0.0188	−1.452 ± 1.033	1.011 Inf	5.846 ± 0.6013	4.745 ± 0.6784	+	No	Medium	High	0.8383 ± 0.2339
10	0.05669 ± 0.0276	0.02327 ± 0.0235	−1.356 ± 1.033	1.428 ± 0.621	5.838 ± 0.6013	4.194 ± 0.6945	+	No	Medium	High	0.8663 ± 0.2213
11	0.1106 ± 0.1092	0.0545 ± 0.0934	−0.1336 ± 0.7036	1.769 ± 0.621	5.596 ± 0.6013	4.113 ± 0.6215	+	No	Medium	High	0.9996 ± 0.0143
12	0.116 ± 0.1045	0.05277 ± 0.0851	−0.2071 ± 0.7036	1.733 ± 0.621	5.531 ± 0.6013	4.046 ± 0.6289	+	No	High	High	0.9999 ± 0.0064
13	0.1043 ± 0.1185	0.05485 ± 0.0875	0.2773 ± 1.033	0.5174 ± 0.621	5.472 ± 0.6013	5.641 ± 0.5563	+	Yes	Medium	High	0.9986 ± 0.02644
14	0.1803 ± 0.1915	0.1212 ± 0.1695	0.5908 ± 1.033	1.591 ± 0.621	5.424 ± 0.6013	4.599 ± 0.6128	+	Yes	Medium	High	0.9992 ± 0.02006
15	0.09613 ± 0.0996	0.05569 ± 0.0867	0.1177 ± 1.033	1.665 ± 0.621	5.624 ± 0.6013	4.759 ± 0.6384	+	Yes	Medium	High	0.9807 ± 0.09642
16	0.1215 ± 0.1321	0.05517 ± 0.0957	−0.1477 ± 1.033	1.112 ± 0.621	5.538 ± 0.6013	5.524 ± 0.6324	+	Yes	High	High	1 ± 0.004919
17	0.06576 ± 0.0682	0.03158 ± 0.0509	−0.00941 ± 1.033	0.4516 ± 0.621	5.695 ± 0.6013	5.643 ± 0.5695	+	Yes	Medium	High	0.9787 ± 0.1009
18	0.2232 ± 0.2218	0.1406 ± 0.1865	0.6316 ± 1.033	1.975 ± 0.621	5.586 ± 0.6013	4.657 ± 0.6233	+	Yes	High	High	0.9999 ± 0.00568

log S: Prediction of water solubility.

2C9 *p*K*i*: Prediction of metabolism based on the cytochrome model.

hERG *p*IC_*50*_: Prediction of inhibition potential of hERG (contributes to the electrical activity of the heart).

HIA: Prediction of human intestinal absorption.

P-gp: Prediction of compounds as substrates for the permeability glycoprotein.

2D6 affinity: Prediction of affinity based on the cytochrome model.

**TABLE 3:** The toxicity prediction data of the designed hybrids, 7-18.

Compound code	Toxicity prediction							
	OSIRIS property explorer				LAZAR			
	Mutagenic	Tumorigenic	Irritant	Reproductive effect	Maximum daily dose (human) (mmol/kg-b.w./day)	Mutagenicity	Acute toxicity LC_*50*_ (mM)	Carcinogenic potency
7	No	NR	NR	NR	0.00707	Inactive	0.01036	Non-carcinogenic
8	No	NR	NR	NR	0.01096	Inactive	0.00945	Non-carcinogenic
9	Medium risk	NR	NR	NR	0.00611	Inactive	0.00396	Non-carcinogenic
10	No	NR	NR	NR	0.00574	Inactive	0.00311	Non-carcinogenic
11	No	NR	NR	NR	0.00958	Inactive	0.00446	Non-carcinogenic
12	No	NR	NR	NR	0.00692	Inactive	0.00803	Non-carcinogenic
13	No	NR	NR	NR	0.01317	Inactive	0.00650	Non-carcinogenic
14	No	NR	NR	NR	0.02293	Inactive	0.00703	Non-carcinogenic
15	Medium risk	NR	NR	NR	0.02290	Inactive	0.00598	Non-carcinogenic
16	No	NR	NR	NR	0.01675	Inactive	0.00688	Non-carcinogenic
17	No	NR	NR	NR	0.01387	Inactive	0.00548	Non-carcinogenic
18	No	NR	NR	NR	0.02293	Inactive	0.00423	Non-carcinogenic

NR, no risk.

### 4.4 Chemistry

**FIGURE 8:**
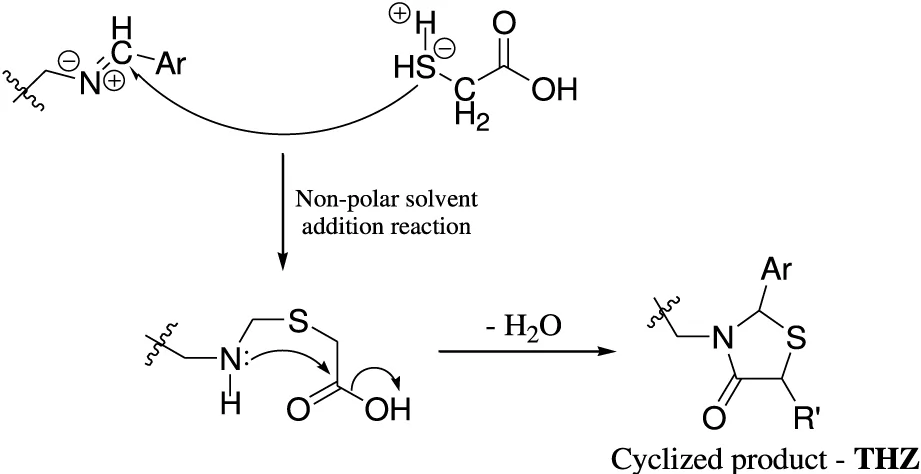
The non-polar solvent addition reaction.

The new hybrids**,** 7-18, were synthesized using the synthetic method as given in Scheme 1. The reaction tends to be more facile due to the presence of a more electronegative group, chlorine, on DCQ, which helps ease the nucleophilic attack by the electrophile ‘NH_2_’ of the used diamine. The diamino-quinoline thus formed was reacted with ten times the excess of the aldehydes, in the presence of a catalytic amount of piperidine, to give the corresponding Schiff bases. The Schiff bases then reacted with thioacids, viz. mercapto-acetic acid and mercapto-succinic acid, upon refluxing with a non-polar solvent such as dioxane, to undergo cycloaddition reaction (Figure 8), thereby yielding the title compounds **7–18**. Here, due to the order of nucleophilicity being C < N < O < S, the formation of compounds would probably take place by an attack of S^−^ on the more electrophilic carbon of Schiff bases, followed by elimination of a molecule of water.

The spectral characterization of the synthesized compounds displayed absorption bands in the IR spectra in the regions corresponding to N-H stretching (3500–3300 cm^-1^), O-H stretching (3400–2400 cm^-1^), C=O stretching (1730–1700 cm^-1^) for carboxylic acid, C=O stretching (1745–1715 cm^-1^) for cyclic ketone, and N-H bending (1500 cm^-1^) for secondary amine. The molecular peak is the parent peak and represents the molecular ion in pure substances. The molecular ion peak of the synthesized compounds in the mass spectrum confirmed the synthesis.

The ^1^H NMR spectra of the compounds showed a singlet at 8.98–8.92 ppm for the secondary amino group, a doublet at 6.11–6.34 ppm for quinoline, and a singlet at 4.36–5.47 ppm for the THZ. All other aliphatic, aromatic, and heteroaromatic protons were found at their predicted ppm values. The characteristics of the compounds were found to be in accordance with the proposed structures. The elemental analysis showed C, H, and N in their intended amounts.

### 4.5 Biological activity

**FIGURE 9:**
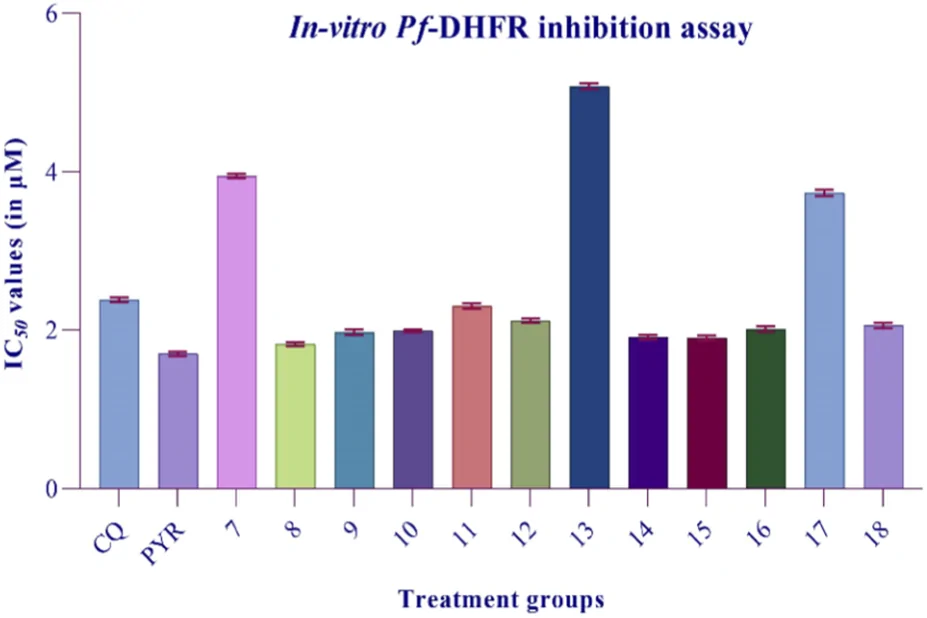
The IC_*50*_ values of the synthesized compounds, 7-18.

The DHFR is a ubiquitous enzyme and plays a key role in the synthesis of thymidine by catalysing the reduction of 7,8-dihydrofolate to 5,6,7,8-tetrahydrofolate with NADPH as the cofactor. The reaction is an essential step in the biosynthesis of nucleotide bases utilized in DNA formation. Inhibition of the enzyme by some molecules, particularly blocking it through a competitive mode of inhibition, will result in cell death due to inhibition of DNA synthesis. If the same happens in a protozoal cell, it will hamper its growth, finally leading to its death. Therefore, *Pf*-DHFR is considered an excellent target for anti-malarial drugs. The potential of the compounds was calculated in terms of IC_*50*_ values as determined from *Pf*-DHFR enzyme inhibition assay (Figure 9). The compounds 8, 9, 14, and 15 exhibited promising potential with IC50 values of 1.82 *μ*M, 1.97 *μ*M, 1.91 *μ*M, and 1.90 *μ*M, which were comparable to that of the reference drug, Pyrimethamine, with IC_*50*_ values of 1.70 *μ*M. Further, the *in-vivo* results confirmed the schizonticidal action of the tested compounds. The percentage parasitaemia inhibition as calculated from Peter’s 4-day suppressive model (Table 4) showed the values quite close to the standard drug, CQ, and ART. The compounds 8, 9, 14, and 15 displayed % PI of 84%, 69%, 82%, and 83%, respectively, with MST of 27 to 23 days. It can be observed that the *in-vitro* and *in-vivo* experiments show concordant results with each other, and with the docking scores collected from the *in silico* analysis.

**TABLE 4:** *In vivo* experimental data of the synthesized hybrids, 7-18.

Comp. code	Group number	Dose (mg/kg b.w.)	% P (mean ± SEM)	% PI (mean ± SEM)	MST (in days) (Mean ± SEM)
Control	1	-	8.51 ± 0.04	-	9.24 ± 0.64
CQ	2	8	0.20 ± 0.01	97.76 ± 0.03	All alive
ART	3	5	0.09 ± 0.01	98.82 ± 0.01	All alive
8	4	50	0.39 ± 0.02	83.39 ± 0.20	26.91 ± 0.64
	5	100	0.30 ± 0.01	84.28 ± 0.18	26.98 ± 0.54
9	6	50	2.68 ± 0.02	68.05 ± 0.22	22.19 ± 0.19
	7	100	2.54 ± 0.01	69.79 ± 0.12	23.48 ± 0.30
14	8	50	1.76 ± 0.03	79.03 ± 0.35	24.29 ± 0.38
	9	100	1.28 ± 0.02	82.71 ± 0.29	25.24 ± 0.25
15	10	50	1.57 ± 0.03	81.30 ± 0.30	24.59 ± 0.44
	11	100	1.17 ± 0.03	83.10 ± 0.27	23.65 ± 0.92

#### 4.5.1 Structure-activity relationship

The synthesized compounds contain two rigid segments in their molecular structure contributed by two hydrophobic moieties, a 4-amino-7-chloroquinoline (4-AQ) and a substituted thiazolidin-4-one ring (THZ), and a flexible segment consisting of a carbon-linker with three carbon atoms. In the other series of the synthesized compounds, the molecule contained the rigid segments without any linker. Since literature surveys suggest the 4-AQ segment to be essential for a compound to exhibit anti-malarial activity, no substitution was made over 4-AQ. The modifications were largely made by introducing substituents at positions two and five of THZ. The experimental results suggested a correlation with the *in silico* data, thereby leading to some definite conclusions.

The quinoline nucleus with a chloro-group at position-7 may be considered a crucial moiety, having been linked to essential amino acid residues in the computational analysis. This may be attributed to the electron-withdrawing effect of the group, which reinforced *pi-pi* interaction with the key amino acid residues. The findings derived from the experimental study corroborated the same conclusion. Additionally, the presence of a carboxylic acid substituent at position five of the 4-THZ was found to be significant and influenced the biological potential of the compounds. With percent parasitaemia inhibition ranging from 83% to 69%, these compounds were shown to have higher schizonticidal action. Additionally, they exhibited IC_*50*_ values in the *Pf*-DHFR enzyme inhibition experiment as low as 1.82 *µ*M. A plausible explanation can be the interaction between the negatively charged carboxyl group and the positively charged regions of the amino group at the receptor site. Moreover, the compounds devoid of a carbon chain-linker exhibited superior efficacy as schizonticides and DHFR inhibitors relative to those with a linker. This is likely attributable to the molecular size of the compounds and their orientation at the target site. Also, the structural component of the most active compounds was either a bicyclic heteroaromatic ring system, viz., 8 and 9, or an aromatic system, either substituted or unsubstituted, featuring a carbon-chain linker and a carboxy substituent at the THZ ring, viz., 14 and 15, which might inculcate the ability of the molecule to interact with the hydrophobic pockets of the active site.

## 6 Conclusion

Malaria, although prevalent, remains a low-priority research domain. Even decades after the introduction of Artemisinin, there has been little development in the field of anti-malaria drugs. Further, the development of resistance against the existing chemotherapy aggravates the situation and calls for the need for the development of novel and effective drug molecules. Literature suggests the 4-aminoquinoline (4-AQ) pharmacophore as the most significant among quinolines. It inhibits plasmodium growth by complexing with ferriprotoporphyrin IX (FPIX) and preventing hemozoin production. The basic amino group in the pharmacophore side chain traps drugs in the parasite’s acidic DV, maintaining high drug concentrations. Additionally, the rigid aryl moiety linked to the 4-AQ is reported to bind *Pf*-DHFR efficiently. The study focuses on the strategies of molecular hybridization and polypharmacology involving the use of CSA to design and synthesize novel 4-aminoquinoline-thiazolidin-4-one hybrids as anti-malarial agents. The primary structural requirement for CSAs is the presence of two aromatic hydrophobic sites with a hydrogen bond acceptor over the nitrogen atom of the side chain of the CSA, with nitrogen itself being secondary or tertiary. The basicity of nitrogen, lipophilicity, and the frontier orbital energies are correlated with the activity of CSAs. Among various nitrogen-containing heterocyclic scaffolds, the thiazolidin-4-ones (THZ) possess adequate characteristics to be regarded as a CSA in antimalarial therapy. They prevent enzymatic dealkylation of the quinoline without affecting its lipophilicity, thereby increasing its antimalarial potential.

The molecules selected from the computational study were synthesized and characterized using physicochemical and spectral techniques. The compounds were subjected to pharmacological screening using *in-vitro* and *in-vivo* methods. They displayed good activity as evidenced by the results of *in-vitro Pf*-DHFR enzyme inhibition assay, with an IC_*50*_ value as low as 1.82 *µ*M. Also, the results from *in-vivo* Peter’s 4-day suppressive test on the selected actives suggest the compounds to be potent, with percentage parasitaemia inhibition to the extent of 84%, and the mean survival time up to 27 days. The results obtained from experimental studies and the *in silico* screening were found to be in sync. Also, the MD simulation study supports the *in silico* molecular docking study, as it showed the stability of the ligand-protein complex up to 200 ns. Further, the *in silico* ADMET prediction data, along with the acute toxicity results, suggest that the tested compounds possess no major toxicity.

Based on the findings of the study, the synthesized compounds are considered to be prospective antimalarial agents. These compounds were designed using the approach of molecular hybridization, involving a chemosenstitizing agent. The effectiveness of the method was established from the experiments, which showed that the compounds exhibited potential not only as DHFR inhibitors but also as schonticides. Thus, it may be concluded that the compounds are capable of functioning as dual inhibitors, and may provide some insight into addressing the issue of resistance development. As a result, the scaffold also has the potential to serve as a lead for the development of new-generation molecules that are effective in treating malaria.

## Data Availability

The original contributions presented in the study are included in the article/Supplementary Material, further inquiries can be directed to the corresponding author.
