# Supplementary material for: Design and syntheses of 4-aminoquinoline-thiazolidinone hybrids as anti-protozoal agents
Source: Front Chem. 2026 Sep 7;14:1892807. doi: 10.3389/fchem.2026.1892807 (PMC13590687; doi:10.3389/fchem.2026.1892807)
Supplement: Supplementary file 1 [file DataSheet1.docx]

**Design and Syntheses of 4-Aminoquinoline-Thiazolidinone Hybrids as Anti-Protozoal Agents**

Gul Naz Fatima^1^, Sarvesh K. Paliwal^2^, Shailendra K. Saraf^1*^

^1^Division of Pharmaceutical Chemistry, Faculty of Pharmacy, Babu Banarasi Das Northern India Institute of Technology, Lucknow, Uttar Pradesh, 226028, India.

^2^Department of Pharmacy, Banasthali Vidyapith, Banasthali, Tonk, Rajasthan, 304022, India.

*Corresponding author:

Prof. (Dr.) Shailendra K. Saraf

E-mail address: [dirpharmniec@gmail.com](mailto:dirpharmniec@gmail.com)

Phone number: +919839228022

**Supplementary Material**

**S1:** IR, Mass, ^1^H-NMR and ^13^C-NMR spectra of the synthesized compounds, 7-18.

IR spectrum of the synthesized compound 7

Mass spectrum of the synthesized compound 7

^1^H-NMR spectrum of the synthesized compound 7

IR spectrum of the synthesized compound 8

Mass spectrum of the synthesized compound 8

^1^H-NMR spectrum of the synthesized compound 8

^13^C-NMR spectrum of the synthesized compound 8

IR spectrum of the synthesized compound 9

Mass spectrum of the synthesized compound 9

^1^H-NMR spectrum of the synthesized compound 9

^13^C-NMR spectrum of the synthesized compound 9

IR spectrum of the synthesized compound 10

Mass spectrum of the synthesized compound 10

^1^H-NMR spectrum of the synthesized compound 10

^13^C-NMR spectrum of the synthesized compound 10

IR spectrum of the synthesized compound 11

Mass spectrum of the synthesized compound 11

^1^H-NMR spectrum of the synthesized compound 11

^13^C-NMR spectrum of the synthesized compound 11

IR spectrum of the synthesized compound 12

Mass spectrum of the synthesized compound 12

^1^H-NMR spectrum of the synthesized compound 12

^13^C-NMR spectrum of the synthesized compound 12

IR spectrum of the synthesized compound 13

Mass spectrum of the synthesized compound 13

^1^H-NMR spectrum of the synthesized compound 13

^13^C-NMR spectrum of the synthesized compound 13

IR spectrum of the synthesized compound 14

Mass spectrum of the synthesized compound 14

^1^H-NMR spectrum of the synthesized compound 14

^13^C-NMR spectrum of the synthesized compound 14

IR spectrum of the synthesized compound 15

Mass spectrum of the synthesized compound 15

^1^H-NMR spectrum of the synthesized compound 15

^13^C-NMR spectrum of the synthesized compound 15

IR spectrum of the synthesized compound 16

Mass spectrum of the synthesized compound 16

^1^H-NMR spectrum of the synthesized compound 16

^13^C-NMR spectrum of the synthesized compound 16

IR spectrum of the synthesized compound 17

Mass spectrum of the synthesized compound 17

^1^H-NMR spectrum of the synthesized compound 17

^13^C-NMR spectrum of the synthesized compound 17

IR spectrum of the synthesized compound 18

Mass spectrum of the synthesized compound 18

^1^H-NMR spectrum of the synthesized compound 18

^13^C-NMR spectrum of the synthesized compound 18

**S2:** The copy of the Institutional Animal Ethics Committee (IAEC) approval certificate.

**S3:** Number of infected RBCs per five thousand RBCs counted.

| **Comp Code** | **Group Number** | **No. of infected RBCs** | | | | |
| --- | --- | --- | --- | --- | --- | --- |
|  |  | **I** | **II** | **III** | **IV** | **V** |
| Control | 1 | 429 | 428 | 419 | 424 | 427 |
| CQ | 2 | 9 | 8 | 10 | 11 | 12 |
| ART | 3 | 5 | 3 | 6 | 5 | 4 |
| 8 | 4 | 19 | 22 | 21 | 18 | 18 |
|  | 5 | 14 | 16 | 15 | 13 | 17 |
| 9 | 6 | 133 | 132 | 137 | 136 | 131 |
|  | 7 | 126 | 125 | 128 | 129 | 126 |
| 14 | 8 | 87 | 85 | 91 | 92 | 86 |
|  | 9 | 67 | 65 | 63 | 62 | 64 |
| 15 | 10 | 77 | 75 | 81 | 82 | 78 |
|  | 11 | 57 | 54 | 61 | 62 | 58 |

**S4:** Survival time (in days) of the individual animals in the group.

| **Comp Code** | **Group Number** | **Survival time in no. of days** | | | | |
| --- | --- | --- | --- | --- | --- | --- |
|  |  | **I** | **II** | **III** | **IV** | **V** |
| Control | 1 | 7.31 | 8.48 | 9.56 | 9.75 | 11.09 |
| CQ | 2 | -NA- | | | | |
| ART | 3 | -NA- | | | | |
| 8 | 4 | 26.18 | 27.2 | 27.25 | 27.5 | 26.4 |
|  | 5 | 27.25 | 27.50 | 27.50 | 24.84 | 27.80 |
| 9 | 6 | 21.68 | 22.68 | 22.40 | 21.80 | 22.40 |
|  | 7 | 22.50 | 23.50 | 23.50 | 23.50 | 24.40 |
| 14 | 8 | 23.45 | 24.42 | 25.50 | 24.60 | 23.50 |
|  | 9 | 24.50 | 25.20 | 25.50 | 26.00 | 25.00 |
| 15 | 10 | 24.48 | 25.44 | 25.52 | 24.43 | 23.10 |
|  | 11 | 21.40 | 21.45 | 25.50 | 25.40 | 24.50 |
